# Undiagnosed Diabetes in Breast, Colorectal, Lung, and Prostate Cancer: Incidence and Risk Factors

**DOI:** 10.1155/2014/607850

**Published:** 2014-03-04

**Authors:** Robert I. Griffiths, Karla J. Lindquist, Cynthia D. O'Malley, Michelle L. Gleeson, Jennifer L. Duryea, José M. Valderas, Mark D. Danese

**Affiliations:** ^1^Nuffield Department of Primary Care Health Sciences, University of Oxford, 23-38 Hythe Bridge Street, 2nd Floor, Oxford OX1 2ET, UK; ^2^Division of General Internal Medicine, Johns Hopkins University School of Medicine, Baltimore, MD 21205, USA; ^3^Department of Epidemiology, Outcomes Insights, Inc., Westlake Village, CA 91362, USA; ^4^Center for Observational Research, Amgen Inc., Thousand Oaks, CA 91320, USA

## Abstract

Our study describes the incidence and risk factors for undiagnosed diabetes in elderly cancer patients. Using Surveillance, Epidemiology, and End Results-Medicare data, we followed patients with breast, colorectal, lung, or prostate cancer from 24 months before to 3 months after cancer diagnosis. Medicare claims were used to exclude patients with diabetes 24 to 4 months before cancer (look-back period), identify those with diabetes undiagnosed until cancer, and construct indicators of preventive services, physician contact, and comorbidity during the look-back period. Logistic regression analyses were performed to identify factors associated with undiagnosed diabetes. Overall, 2,678 patients had diabetes undiagnosed until cancer. Rates were the highest in patients with both advanced-stage cancer and low prior primary care/medical specialist contact (breast 8.2%, colorectal 5.9%, lung 4.4%). Nonwhite race/ethnicity, living in a census tract with a higher percent of the population in poverty and a lower percent college educated, lower prior preventive services use, and lack of primary care and/or medical specialist care prior to cancer all were associated with higher (*P* ≤ 0.05) adjusted odds of undiagnosed diabetes. Undiagnosed diabetes is relatively common in selected subgroups of cancer patients, including those already at high risk of poor outcomes due to advanced cancer stage.

## 1. Introduction

Diabetes and the metabolic derangements typical of diabetes are associated with poor prognosis in cancer [1–11]. In perhaps the most comprehensive study to date; Barone and colleagues [2] performed a systematic review and meta-analysis of the literature and found that preexisting diabetes was associated with statistically significant increases of 41% for all-cause mortality, across multiple tumor types, and 76%, 61%, and 32% in endometrial, breast, and colorectal cancer, respectively. Poor prognosis may be influenced through biological mechanisms related to hyperglycemia, hyperinsulinemia, and inflammation, which result in tumor cell proliferation and metastases [3–5, 12]. Other factors include less aggressive cancer treatment due to diabetes-related comorbidity [13], poorer response to cancer treatment [7, 11], presentation with later-stage cancer due to suboptimal cancer screening practices and other preventive health-seeking behavior [14], and that diagnosis of cancer may distract both the patient and the health care team from appropriate management of glycemia, blood pressure, and lipids [2].

Factors thought to play a role in observed associations between preexisting diabetes and mortality [2] could be exacerbated in undiagnosed diabetes, but evidence supporting this hypothesis is scarce. Data from the Second National Health and Nutrition Examination Survey (NHANES) do suggest that cancer mortality in patients with undiagnosed diabetes may be higher than in those with previously diagnosed diabetes, where undiagnosed diabetes was identified by oral glucose tolerance testing [9]. However, this study was conducted in the general population making the risk of cancer mortality a function of both the risk of developing cancer and the subsequent risk of death due to cancer. Also, the two diabetes groups were not compared directly, and differences in mortality compared to a reference group with normal glucose tolerance, while suggestive of an adverse impact, failed to reach statistical significance [9].

Data on the incidence of and risk factors for undiagnosed diabetes in cancer also are scarce. While a recent paper reports that detection of many chronic conditions—including diabetes—increases around the time of breast cancer diagnosis [15], risk factors for the detection of these conditions were not examined in detail. Several studies have examined factors associated with cancer stage at diagnosis, an important predictor of cancer mortality, focusing on demographic and socioeconomic characteristics and patterns of prior health system contact [16–19]. These show that more contact with a primary care physician and/or medical specialist [16], greater use of general preventive and cancer screening services [17], and more contact with the health care system as measured by level of comorbidity ascertained through medical claims [18], all are associated with earlier-stage cancer at diagnosis.

In this study, we sought to describe the incidence and risk factors for diabetes that is undiagnosed until cancer. We elected to focus on prior health system contact, comorbidity, race, and socioeconomic status as risk factors since there is evidence that all of these are associated with cancer stage at diagnosis [16–19], which is another important prognostic factor for cancer outcomes.

## 2. Methods

### 2.1. Data Source

The source of data for this study was the National Cancer Institute's (NCI) Surveillance, Epidemiology, and End Results (SEER) cancer registry linked to Medicare claims [20]. Presently, SEER contains cancer incidence and survival data from 17 population-based cancer registries throughout the United States covering approximately 28% of the population [21]. In SEER-Medicare, cancer registry data are linked to Medicare enrollment and claims data, which are available for 93% of those aged ≥65 years in the SEER registry [22].

### 2.2. Inclusion and Exclusion Criteria

Patients meeting all of the following criteria were included: they were diagnosed with breast, colorectal, lung, or prostate cancer, the four most common types in the elderly [23], between January 1, 1999, and December 31, 2002. This was their first and only cancer diagnosed, and they had at least 24 months of Medicare Part A (hospital) and Part B (outpatient) fee-for-service coverage prior to the diagnosis of cancer. Patients were excluded for the following reasons: male breast cancer, cancer diagnosis made by death certificate or autopsy, death within the first month following diagnosis, missing or unknown cancer stage at diagnosis, or *in situ* lung or prostate cancer (due to small numbers of patients).

Patients with preexisting diabetes diagnosed between 24 and 4 months (inclusive) before cancer initially were included in the study to calculate the proportion of all diabetes cases undiagnosed *until* cancer (i.e., using a denominator of preexisting plus undiagnosed until cancer). However, patients with preexisting diabetes were then excluded from all the analyses of risk factors for undiagnosed diabetes. Diabetes was defined as the presence of one of the following International Classification of Diseases, 9th Revision, Clinical Modification diagnosis codes in one inpatient Medicare claim or in two outpatient claims at least 30 days apart: 250.xx for diabetes and complications, 357.2x for polyneuropathy in diabetes, 362.0x for diabetic retinopathy, and 366.41 for diabetic cataract [24]. Laboratory claims were excluded to reduce the likelihood of misclassifying those patients only undergoing diagnostic evaluation for suspected diabetes as actual diabetes cases.

### 2.3. Observation Period

Patients were followed from 24 months prior to the diagnosis of cancer until 3 months after diagnosis (overall follow-up: 27 months). The observation period was divided into two consecutive periods: 24 to 4 months prior to cancer diagnosis (the 21-month look-back period) and 3 months prior to 3 months after diagnosis or until death, whichever came first (the 6-month incidence period). The first day of the SEER month of diagnosis was assigned as the day of diagnosis. The look-back period was used to identify preexisting (prevalent) diabetes and to construct measures of prior health system contact. The incidence period was used to identify previously undiagnosed diabetes.

### 2.4. Definition of Undiagnosed Diabetes

Diabetes that was not reported in the claims until the time around cancer diagnosis was considered to be “undiagnosed diabetes.” It was defined as having a first diagnosis of diabetes between 3 months before and 3 months after cancer diagnosis. The same claims-based algorithm [24] used to identify and subsequently exclude patients with preexisting diabetes during the look-back period (24 to 4 months before cancer diagnosis) also was used to identify undiagnosed diabetes.

### 2.5. Patients and Variables

Patients were described according to their demographic, clinical, and socioeconomic characteristics. Requiring eligible patients to have at least two years of Medicare enrollment prior to diagnosis meant that the minimum age in the cohort was 67 years. Race/ethnicity was defined using the SEER recoded race variable [25]. Stage at cancer diagnosis was based on the SEER-modified American Joint Committee on Cancer (AJCC) stage variable [25]. In SEER, socioeconomic information, including measures of poverty and education, is reported at the census tract level.

We constructed two measures of prior health system contact during the look-back period, based on literature describing associations between prior health system contact and stage at cancer diagnosis [16, 17]. First, we constructed a physician contact index that classified patients according to the types of ambulatory care visits they received during the look-back period [16]. We searched Medicare claims for Healthcare Common Procedure Coding System codes indicating physician outpatient visits and used the associated physician specialty code to classify each visit as primary care physician, medical specialist, or other specialist. Other specialists included general surgeons, ophthalmologists, orthopedic surgeons, and other surgical specialists [16]. Patients were then classified as having had (A) primary care physician but no medical specialist (with or without other specialist) visits, (B) medical specialist but with no primary care physician (with or without other specialist) visits, (C) both primary care physician and medical specialist visit, (D) other specialist but no primary care physician or medical specialist visits, or (E) no prior visits.

Second, we constructed an index of preventive services based on the one developed by Gornick et al. [17], consisting of mammography, screening for colorectal cancer, prostate-specific antigen test, Papanicolaou test, screening for glaucoma, influenza immunization, and pneumonia immunization. The presence of one or more claims for each type of service was coded as “1” for that service, and individual scores were combined in an index consisting of 0, 1, or ≥2.

Comorbidity is a predictor of breast cancer stage at diagnosis [18], and when comorbidity is identified from medical claims, it can also be considered an indirect indicator of increased health system contact. To account for this facet of health system contact, we calculated an NCI comorbidity index score for each patient [22–32].

### 2.6. Analyses

We calculated the proportion of *all* diabetes patients diagnosed during the entire 27-month observation period who were diagnosed during the 6-month incidence period. We then excluded those with preexisting diabetes from the remainder of the analyses. We performed four multivariate logistic regression analyses, one for each type of cancer, to examine race/ethnicity, socioeconomic factors, and patterns of prior health system contact associated with undiagnosed diabetes—all stratified by type of cancer. All models included age, gender (colorectal and lung only), race/ethnicity, year of diagnosis, education, poverty, and geographic area. All three measures of prior health system contact (physician contact index, index of preventive services, and NCI Comorbidity Index) were included in each of the four models. However, the three measures of prior health system contact also were assessed in separate models to evaluate the levels of multicollinearity among them.

## 3. Results

Initially, 184,336 patients with breast, colorectal, lung, and prostate cancer were considered for inclusion in the study. Of these, 11,426 (6.2%) were excluded due to missing/unknown cancer stage (all cancer types) or *in situ* stage (lung and prostate cancer only, as stated in Section 2). Of the remaining 172,910, an additional 18,218 (10.5%) were excluded prior to the analysis of risk factors for undiagnosed diabetes because they had preexisting diabetes diagnosed during the look-back period: breast 3,850/40,062 (9.6%), colorectal 6,029/39,034 (15.4%), lung 4,861/40,622 (12.0%), and prostate 3,478/53,182 (6.5%). Therefore, 154,692 met all the inclusion and exclusion criteria for the analysis of risk factors associated with undiagnosed diabetes (Table 1). Overall, the mean age was 76.3 years: 84.7% were non-Hispanic white, 31.6% came from a census tract with >12% poverty, and 56.0% were from a large metropolitan area.

Among the measures of prior health system contact, 65.3% had an NCI comorbidity index score equal to 0, 78.1% had at least one preventive service during the look-back period, and 56.5% had visits to both a primary care physician and a medical specialist (Table 2). This differed by cancer type as 69.5% and 69.0% of breast cancer and prostate cancer patients, respectively, had NCI comorbidity index scores of 0 compared to 53.9% of lung cancer patients. Breast (19.2%) and prostate (19.6%) cancer patients were more likely to have had no preventative service visits compared to colorectal (25.2%) or lung (24.8%) cancer patients. Women with breast cancer (60.4%) were more likely to have visited both a primary care physician and medical specialist than were other patients in this study (53.3% to 57.2%).

**Figure 1:**
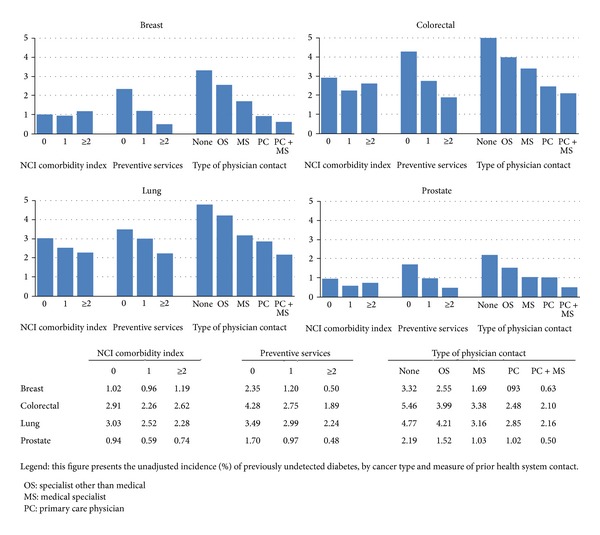
Incidence of undiagnosed diabetes and measure of prior health system contact.

Overall, 2,678 had undiagnosed diabetes (Table 3). When viewed over the entire study period (the incidence and the prevalence look-back periods combined), undiagnosed diabetes accounted for 12.8% (2,678/20,896) of all the diabetes cases: 8.8% of all diabetes in breast, 13.0% of all diabetes in colorectal, 16.8% of all diabetes in lung, and 10.8% of all diabetes in prostate cancer (not shown in the tables). In general, the incidence of undiagnosed diabetes in the 6-month period around the cancer diagnosis was similar across age groups but was higher in those of nonwhite race/ethnicity, those diagnosed with advanced stage cancer, those living in a census tract with a lower proportion college educated, and those living in a census tract with more poverty. The incidence of undiagnosed diabetes was inversely related to the number of preventive services (Figure 1). It was also lower in patients who had visits to a primary care physician and/or a medical specialist. These associations were consistent across the four types of cancer. Rates were highest among those with no outpatient physician care during the look-back period (2.2% in prostate, 5.5% in colorectal, 4.8% lung, and 3.3% breast cancer).

In multivariate analyses that included all three measures of prior health system contact in the same model, the adjusted odds of undiagnosed diabetes were statistically significantly lower for those with 1 or ≥2 preventive services (compared to none) in breast, colorectal, and prostate (≥2 only) but not in colorectal and lung cancer (Table 4). The adjusted odds of undiagnosed diabetes also were lower for those with primary care and/or medical specialist care prior to cancer.

In general, nonwhite race/ethnicity was associated with increased adjusted odds of undiagnosed diabetes, as was living in a census tract with a lower percent college educated and a higher percent in poverty (Table 4). Overall, effect sizes for measures of prior health system contact were larger in models that included only one measure per model (Table 5).

## 4. Conclusions

In this study, we described the epidemiology of undiagnosed diabetes in a large cohort of elderly cancer patients in the United States. Our findings show that undiagnosed diabetes accounted for almost 13% of all diabetes cases identified in an older cohort of patients diagnosed with cancer. This proportion is lower than the one obtained from the general population sampled in NHANES [9], in which participants underwent an oral glucose tolerance test and the results were compared to self-reported history of diabetes to classify patients as previously diagnosed or undiagnosed. In that study, compared to the group with normal glucose tolerance, those with undiagnosed diabetes were more likely to be nonwhite race/ethnicity and to have less than a high school education. In this regard, our findings were similar in this cohort of elderly cancer patients, all of whom had at least two years of health insurance prior to cancer diagnosis.

Furthermore, we found that the highest rates of undiagnosed diabetes were observed in those with limited health system contact prior to cancer and in those with advanced-stage cancer. Previous research shows that limited health system contact is associated with advanced-stage cancer at diagnosis [16–18]. However, the fact that limited health system contact is associated with *both* advanced stage and undiagnosed diabetes does not rule out other mechanisms, for example, biological, linking undiagnosed diabetes directly to cancer stage. In addition, in unadjusted models, and several of the adjusted models, both higher levels of poverty and lower levels of education were associated with a greater likelihood of diabetes being diagnosed in the period around cancer diagnosis. This suggests that socioeconomic factors are contributors to undiagnosed diabetes. The fact that models with all risk factors show some attenuation of the socioeconomic factors suggests that interaction with the healthcare system and socioeconomic status are confounded.

The association between undiagnosed diabetes and cancer prognosis is complex. Plausible mechanisms include exacerbated biological effects [3–5] and the added burden on the health care team of managing a previously undiagnosed condition [2], which could impact treatment selection [12] and response [7, 11]. Nevertheless, our findings indicate that those already at the greatest risk of poor cancer outcomes due to advanced stage also are most likely to bear any additional adverse prognostic burden of undiagnosed diabetes.

Our study has several limitations. The approach to identifying undiagnosed diabetes entailed first dividing an observation period from 24 months before to 3 months after cancer diagnosis into a look-back period and an incidence period, then using a claims-based algorithm [24] to identify diabetes in each period, and finally excluding those with preexisting diabetes from the incidence risk set. The claims-based algorithm we used has a sensitivity of 70% [24]. Also, in our study, the overall prevalence of diabetes during the observation period (look-back and incidence periods combined) was lower than that previously reported in the literature based, for instance, on hospital medical records review [13]. Consequently, it is likely that we have also underestimated the incidence of undiagnosed diabetes.

Also, to preserve a large sample and include patients as close to the minimum age for Medicare eligibility (65 years) as possible, we established the beginning of the look-back period at 24 months before cancer diagnosis, thereby making the minimum age at cancer diagnosis 67 years in this cohort. Many of these patients had a claims history beginning more than 24 months before cancer. However, we elected not to use these in identifying preexisting diabetes because this may have indirectly biased the association between patient age at cancer diagnosis and the incidence of undiagnosed diabetes. Extending the look-back period farther back in time could have resulted in detecting and excluding more cases of preexisting diabetes and possibly also reducing misclassification of preexisting diabetes as undiagnosed cases during the incidence period, but that would have resulted in smaller sample sizes and the exclusion of younger patients. Furthermore, following precedent [9], we have described diabetes first detected during the incidence period as “undiagnosed,” which implies that it was present but undetected prior to that. However, simply by chance, it is likely that some of these cases became diabetic during the incidence period. Therefore, in this study, we may have underestimated the magnitude and statistical significance of associations between prior health system contact and undiagnosed diabetes.

This study was conducted prior to the implementation of the Medicare Modernization Act (MMA), which introduced new coverage for diabetes and other screening services in 2005 [33]. Introduction of these services is designed to improve early detection of diabetes and other important conditions. Therefore, rates of undiagnosed diabetes could have changed due to the implementation of the MMA. In addition to affecting the incidence of undiagnosed diabetes, the MMA would impact services included in the preventive services measure of prior health system contact. Since some of the new services impact diabetes, it is possible that associations between level of preventive services use and undiagnosed diabetes would become stronger as a result but that the overall incidence of undiagnosed diabetes would have declined through improved coverage of preventive services.

Limitations notwithstanding, our findings indicate that undiagnosed diabetes is relatively common in selected subgroups of cancer patients, such as those with limited prior health system contact and advanced cancer stage, and that poverty and lower educational attainment may contribute directly, as well as through limited interaction with the health system. Also, those already at the greatest risk of poor cancer outcomes due to advanced stage also are most likely to bear any additional adverse prognostic burden of undiagnosed diabetes. Possible explanations for the association between undiagnosed diabetes and advanced stage include biological mechanisms and/or shared risk factors. As the incidence of diabetes continues to rise, understanding the relationship between undiagnosed diabetes and cancer outcomes may help inform treatment decisions in the management of these patients.

## Figures and Tables

**Table 1:** Patient characteristics.

	Overall		Type of cancer							
			Breast		Colorectal		Lung		Prostate	
	*N*	% (SD)	*n*	% (SD)	*n*	% (SD)	*n*	% (SD)	*n*	% (SD)
	154,692	100	36,212	23.4	33,005	21.3	35,761	23.1	49,714	32.1
Age at diagnosis (years)										
67–70	**23,498**	**15.2**	5,437	15.0	3,661	11.1	5,339	14.9	9,061	18.2
71–75	**44,578**	**28.8**	9,936	27.4	7,503	22.7	10,631	29.7	16,508	33.2
76–80	**41,887**	**27.1**	9,630	26.6	8,545	25.9	10,090	28.2	13,622	27.4
>80	**44,729**	**28.9**	11,209	30.1	13,296	40.3	9,701	27.1	10,523	21.2
Mean and (SD) age	**76.3**	**(6.7)**	76.6	(6.5)	78.1	(6.8)	76.0	(6.0)	75.1	(7.0)
Gender										
Male	**83,272**	**53.8**	NI	NI	14,739	44.7	18,819	52.6	49,714	100
Female	**71,420**	**46.2**	36,212	100	18,266	55.3	16,942	47.4	NA	NA
Race/ethnicity										
White	**131,000**	**84.7**	31,923	88.2	28,162	85.3	30,824	86.2	40,091	80.6
Black	**11,227**	**7.3**	2,048	5.7	2,158	6.5	2,609	7.3	4,412	8.9
Hispanic	**5,374**	**3.5**	1,066	2.9	1,182	3.6	987	2.8	2,139	4.3
Other	**7,091**	**4.6**	1,175	3.2	1,503	4.6	1,341	3.8	3,072	6.2
Year of diagnosis										
1999	**21,578**	**14.0**	5,300	14.6	4,542	13.8	4,527	12.7	7,209	14.5
2000	**44,195**	**28.6**	10,283	28.4	9,701	29.4	10,421	29.1	13,790	27.7
2001	**44,927**	**29.0**	10,517	29.0	9,486	28.7	10,613	29.7	14,311	28.8
2002	**43,992**	**28.4**	10,112	27.9	9,276	28.1	10,200	28.5	14,404	29.0
Stage at diagnosis										
* In situ *	**8,456**	**8.1**	5,697	15.7	2,759	8.4	NI	NI	NI	NI
I	**34,001**	**32.4**	16,493	45.6	8,635	26.2	8,873	24.8	NA	NA
II	**21,374**	**20.4**	10,565	29.2	9,575	29.0	1,234	3.5	NA	NA
III	**20,737**	**19.8**	1,852	5.1	7,195	21.8	11,690	32.7	NA	NA
IV	**20,410**	**19.4**	1,605	4.4	4,841	14.7	13,964	39.1	NA	NA
Localized	**47,244**	**95.0**	NA	NA	NA	NA	NA	NA	47,244	95.0
Distant	**2,470**	**5.0**	NA	NA	NA	NA	NA	NA	2,470	5.0
Percent in census tract with some college*										
<25%	**53,892**	**34.8**	11,975	33.1	12,016	36.4	12,690	35.5	17,211	34.6
≥25%	**100,781**	**65.2**	24,233	66.9	20,984	63.6	23,067	64.5	32,497	65.4
Percent in census tract living in poverty										
<5%	**48,053**	**31.1**	11,773	32.5	9,935	30.1	9,928	27.8	16,417	33.0
5–7%	**21,425**	**13.9**	5,366	14.8	4,580	13.9	4,739	13.3	6,740	13.6
8–12%	**34,511**	**22.3**	8,226	22.7	7,538	22.8	8,049	22.5	10,698	21.5
>12%	**48,929**	**31.6**	10,428	28.8	10,511	31.9	12,689	35.5	15,301	30.8
Missing	**1,774**	**1.2**	419	1.2	441	1.3	356	1.0	558	1.1
Type of geographic area										
Large metropolitan	**86,555**	**56.0**	20,678	57.1	18,383	55.7	19,759	55.3	27,735	55.8
Metropolitan	**42,693**	**27.6**	10,139	28.0	9,077	27.5	9,686	27.1	13,791	27.7
Urban	**9,799**	**6.3**	2,206	6.1	2,084	6.3	2,444	6.8	3,065	6.2
Less urban/rural	**15,645**	**10.1**	3,189	8.8	3,461	10.5	3,872	10.8	5,123	10.3

SD: standard deviation; NI: not included in the study; NA: not applicable; *19 patients had a missing value.

**Table 2:** Measures of prior health care system contact.

	Overall		Type of cancer							
			Breast		Colorectal		Lung		Prostate	
	*N*	%	*n*	%	*n*	%	*n*	%	*n*	%
	154,692	100	36,212	23.4	33,005	21.3	35,761	23.1	49,714	32.1
NCI comorbidity index										
0	**100,929**	**65.3**	25,174	69.5	22,151	67.1	19,284	53.9	34,320	69.0
1	**34,778**	**22.5**	7,582	20.9	6,917	21.0	9,890	27.7	10,389	20.9
≥2	**18,985**	**12.3**	3,456	9.5	3,937	11.9	6,587	18.4	5,005	10.1
Preventive services										
0	**33,873**	**21.9**	6,937	19.2	8,322	25.2	8,866	24.8	9,748	19.6
1	**40,735**	**26.3**	8,582	23.7	9,567	29.0	9,640	27.0	12,946	26.0
≥2	**80,084**	**51.8**	20,693	57.1	15,116	45.8	17,255	48.3	27,020	54.4
Types of physician visits										
Primary care and medical specialist	**87,436**	**56.5**	21,857	60.4	17,591	53.3	19,573	54.7	28,415	57.2
Primary care, no medical specialist	**22,462**	**14.5**	7,285	20.1	6,577	19.9	6,490	18.2	6,686	13.5
Medical specialist, no primary care	**27,038**	**17.5**	3,839	10.6	4,738	14.4	5,060	14.2	8,825	17.8
Other specialist only	**3,952**	**2.6**	942	2.6	878	2.7	951	2.7	1,181	2.4
None	**13,804**	**8.9**	2,289	6.3	3,221	9.8	3,687	10.3	4,607	9.3

**Table 3:** Incidence of undiagnosed diabetes during 6-month period around cancer diagnosis.

	Overall		Type of cancer							
			Breast		Colorectal		Lung		Prostate	
	*N*	%	*n*	%	*n*	%	*n*	%	*n*	%
	2,678	1.7	370	1.0	904	2.7	983	2.7	421	0.8
Age at diagnosis (years)										
67–70	391	1.66	45	0.83	102	2.79	160	3.00	84	0.93
71–75	753	1.69	94	0.95	218	2.91	318	2.99	123	0.75
76–80	696	1.66	98	1.02	221	2.59	277	2.75	100	0.73
>80	838	1.87	133	1.19	363	2.73	228	2.35	114	1.08
Gender										
Male	1,421	1.71	NI	NI	447	3.03	553	2.94	421	0.8
Female	1,257	1.76	370	1.02	457	2.50	430	2.54	NA	NA
Race/ethnicity										
White	2,091	1.60	289	0.91	705	2.50	779	2.53	318	0.79
Black	300	2.68	50	2.44	94	4.36	109	4.18	47	1.07
Hispanic	144	2.02	15	1.41	49	4.15	45	4.56	35	1.64
Other	143	2.02	16	1.36	56	3.73	50	3.73	21	0.68
Year of diagnosis										
1999	365	1.7	57	1.08	105	2.31	136	3.00	67	0.93
2000	746	1.7	91	0.88	251	2.59	275	2.64	129	0.94
2001	795	1.8	117	1.11	284	2.99	280	2.64	114	0.80
2002	772	1.8	105	1.04	264	2.85	292	2.86	111	0.77
Stage at diagnosis										
*In situ *	77	0.91	22	0.39	55	1.99	NI	NI	NI	NI
I	669	1.24	110	0.67	215	2.94	200	2.25	NA	NA
II	501	1.89	144	1.36	282	2.95	26	2.11	NA	NA
III	602	2.60	38	2.05	227	3.15	314	2.69	NA	NA
IV	690	2.88	56	3.49	125	2.58	443	3.17	NA	NA
Localized	NA	NA	NA	NA	NA	NA	NA	NA	50	2.02
Distant	NA	NA	NA	NA	NA	NA	NA	NA	371	0.79
Percent in census tract with some college										
<25%	1,087	2.02	151	1.26	374	3.11	397	3.13	165	0.96
≥25%	1,590	1.58	219	0.90	529	2.52	586	2.54	256	0.79
Percent in census tract living in poverty										
<5%	Not shown									
5–7%	323	1.51	49	0.91	105	2.29	118	2.49	51	0.76
8–12%	606	1.76	81	0.98	204	2.71	220	2.73	101	0.94
>12%	1,073	2.19	157	1.51	355	3.38	394	3.11	167	1.09
Missing	Not shown									
Type of geographic area										
Large metropolitan	1,513	1.75	204	0.99	512	2.79	566	2.86	231	0.83
Metropolitan	660	1.55	103	1.02	226	2.49	230	2.37	101	0.73
Urban	175	1.79	29	1.31	59	2.83	60	2.45	27	0.88
Less urban/rural	330	2.11	34	1.07	107	3.09	127	3.28	62	1.21

NI: not included in the study; NA: not applicable; not shown: one or more cells contained fewer than 11 observations.

**Table 4:** Multivariate analyses of undiagnosed diabetes (all 3 measures of prior health system contact in each model).

	Type of cancer											
	Breast			Colorectal			Lung			Prostate		
	OR	95% CI		OR	95% CI		OR	95% CI		OR	95% CI	
		Lower	Upper		Lower	Upper		Lower	Upper		Lower	Upper
Age at diagnosis (years)												
67–70	Reference											
71–75	1.18	0.83	1.70	1.16	0.91	1.47	1.05	0.86	1.27	0.88	0.66	1.16
76–80	1.28	0.90	1.84	1.06	0.83	1.35	0.98	0.81	1.20	0.91	0.68	1.23
>80	1.28	0.91	1.81	1.15	0.91	1.45	0.86	0.70	1.06	1.37	1.03	1.82
Gender												
Male	Not applicable			Reference						Not applicable		
Female	Not applicable			0.86	0.75	0.99	0.95	0.83	1.08	Not applicable		
Race/ethnicity												
White	Reference											
Black	1.63	1.17	2.28	1.37	1.08	1.75	1.38	1.11	1.73	0.86	0.61	1.21
Hispanic	1.12	0.65	1.91	1.38	1.02	1.87	1.62	1.18	2.22	1.49	1.03	2.16
Other	1.39	0.83	2.33	1.48	1.11	1.96	1.43	1.06	1.92	0.80	0.50	1.26
Year of diagnosis												
1999	Reference											
2000	0.78	0.56	1.10	1.12	0.89	1.42	0.87	0.71	1.08	0.97	0.72	1.31
2001	0.98	0.71	1.35	1.30	1.03	1.63	0.88	0.72	1.09	0.84	0.62	1.14
2002	0.94	0.67	1.30	1.23	0.97	1.55	0.97	0.78	1.19	0.80	0.59	1.09
Percent in census tract with some college												
<25%	Reference											
≥25%	0.77	0.61	0.95	0.89	0.77	1.02	0.86	0.75	0.99	0.89	0.72	1.10
Percent in census tract living in poverty												
<5%	Reference											
5–7%	1.37	0.95	1.97	0.98	0.77	1.24	1.01	0.81	1.27	1.20	0.85	1.69
8–12%	1.40	1.01	1.94	1.11	0.91	1.35	1.08	0.89	1.31	1.44	1.08	1.92
>12%	1.68	1.24	2.28	1.20	0.99	1.44	1.06	0.86	1.32	1.41	1.06	1.87
Type of geographic area												
Large metropolitan	Reference											
Metropolitan	1.04	0.81	1.34	0.90	0.77	1.07	0.82	0.70	0.96	0.82	0.64	1.04
Urban	1.27	0.84	1.92	1.03	0.77	1.36	0.83	0.63	1.10	0.92	0.69	1.40
less urban/rural	0.89	0.61	1.31	1.04	0.83	1.31	1.07	0.86	1.32	1.10	0.81	1.49
NCI comorbidity index												
0	Reference											
1	1.11	0.85	1.46	0.90	0.75	1.08	0.94	0.80	1.10	0.81	0.61	1.08
≥2	1.28	0.90	1.80	1.01	0.81	1.25	0.88	0.73	1.06	1.02	0.72	1.45
Preventive services												
0	Reference											
1	0.67	0.51	0.87	0.77	0.65	0.92	1.11	0.93	1.33	0.80	0.61	1.04
≥2	0.34	0.26	0.46	0.58	0.48	0.70	0.91	0.76	1.10	0.48	0.36	0.63
Types of physician visits												
None	Reference											
Primary care and medical specialist	0.36	0.26	0.51	0.56	0.45	0.69	52	0.42	0.65	0.41	0.29	0.56
Primary care, no medical specialist	0.43	0.30	0.62	0.59	0.47	0.75	0.64	0.50	0.80	0.67	0.48	0.94
Medical specialist, no primary care	0.74	0.52	1.06	0.78	0.62	0.99	0.73	0.57	0.93	0.69	0.50	0.96
Other specialist only	1.06	0.66	1.71	0.87	0.60	1.27	0.93	0.65	1.33	0.89	0.53	1.49

OR: odds ratio; CI: confidence interval.

**Table 5:** Multivariate analyses of undiagnosed diabetes (each measure of health system contact in a separate model)*.

	Type of cancer											
	Breast			Colorectal			Lung			Prostate		
	OR	95% CI		OR	95% CI		OR	95% CI		OR	95% CI	
		Lower	Upper		Lower	Upper		Lower	Upper		Lower	Upper
NCI comorbidity index												
0	Reference category											
1	0.88	0.67	1.14	0.77	0.64	0.92	0.82	0.71	0.96	0.61	0.46	0.80
≥2	1.02	0.73	1.43	0.88	0.71	1.09	0.75	0.63	0.90	0.74	0.53	1.05
Preventive services												
0	Reference category											
1	0.52	0.41	0.67	0.65	0.56	0.77	0.89	0.76	1.05	0.59	0.47	0.75
≥2	0.24	0.18	0.30	0.46	0.39	0.54	0.68	0.58	0.79	0.31	0.24	0.39
Types of physician visits												
None	Reference category											
Primary care and medical specialist	0.21	0.16	0.28	0.40	0.33	0.49	0.48	0.40	0.58	0.25	0.19	0.33
Primary care, no medical specialist	0.30	0.21	0.41	0.47	0.38	0.58	0.61	0.50	0.76	0.49	0.36	0.68
Medical specialist, no primary care	0.55	0.39	0.78	0.64	0.51	0.79	0.70	0.56	0.87	0.50	0.38	0.68
Other specialist only	0.83	0.52	1.33	0.75	0.52	1.09	0.91	0.64	1.30	0.73	0.44	1.22

*Multivariate models also included age, gender (colorectal and lung cancer only), race/ethnicity, year of diagnosis, education, percent in census tract with some college, percent in census tract living in poverty, and type of geographic area. OR: odds ratio; CI: confidence interval.

## Abbreviations

NHANES: National Health and Nutrition Examination Survey
NCI: National Cancer Institute
SEER: Surveillance, Epidemiology, and End Results
AJCC: American Joint Committee on Cancer
MMA: Medicare Modernization Act.

## References

[1] Giovannucci E, Harlan DM, Archer MC (2010). Diabetes and cancer: a consensus report. *Diabetes Care*.

[2] Barone BB, Yeh H-C, Snyder CF (2008). Long-term all-cause mortality in cancer patients with preexisting diabetes mellitus: a systematic review and meta-analysis. *Journal of the American Medical Association*.

[3] Wolpin BM, Meyerhardt JA, Chan AT (2009). Insulin, the insulin-like growth factor axis, and mortality in patients with nonmetastatic colorectal cancer. *Journal of Clinical Oncology*.

[4] Ma J, Li H, Giovannucci E (2008). Prediagnostic body-mass index, plasma C-peptide concentration, and prostate cancer-specific mortality in men with prostate cancer: a long-term survival analysis. *The Lancet Oncology*.

[5] Lipscombe LL, Goodwin PJ, Zinman B, McLaughlin JR, Hux JE (2008). The impact of diabetes on survival following breast cancer. *Breast Cancer Research and Treatment*.

[6] Richardson LC, Pollack LA (2005). Therapy insight: influence of type 2 diabetes on the development, treatment and outcomes of cancer. *Nature Clinical Practice Oncology*.

[7] Weiser MA, Cabanillas ME, Konopleva M (2004). Relation between the duration of remission and hyperglycemia during induction chemotherapy for acute lymphocytic leukemia with a hyperfractionated cyclophosphamide, vincristine, doxorubicin, and dexamethasone/methotrexate-cytarabine regimen. *Cancer*.

[8] Coughlin SS, Calle EE, Teras LR, Petrelli J, Thun MJ (2004). Diabetes mellitus as a predictor of cancer mortality in a large cohort of US adults. *The American Journal of Epidemiology*.

[9] Saydah SH, Loria CM, Eberhardt MS, Brancati FL (2003). Abnormal glucose tolerance and the risk of cancer death in the United States. *The American Journal of Epidemiology*.

[10] Verlato G, Zoppini G, Bonora E, Muggeo M (2003). Mortality from site-specific malignancies in type 2 diabetic patients from Verona. *Diabetes Care*.

[11] Meyerhardt JA, Catalano PJ, Haller DG (2003). Impact of diabetes mellitus on outcomes in patients with colon cancer. *Journal of Clinical Oncology*.

[12] Wolf I, Sadetzki S, Catane R, Karasik A, Kaufman B (2005). Diabetes mellitus and breast cancer. *The Lancet Oncology*.

[13] van de Poll-Franse LV, Houterman S, Janssen-Heijnen MLG, Dercksen MW, Coebergh JWW, Haak HR (2007). Less aggressive treatment and worse overall survival in cancer patients with diabetes: a large population based analysis. *International Journal of Cancer*.

[14] McBean AM, Yu X (2007). The underuse of screening services among elderly women with diabetes. *Diabetes Care*.

[15] Danese MD, O’Malley C, Lindquist K, Gleeson M, Griffiths RI (2012). An observational study of the prevalence and incidence of comorbid conditions in older women with breast cancer. *Annals of Oncology*.

[16] Keating NL, Landrum MB, Ayanian JZ, Winer EP, Guadagnoli E (2005). The association of ambulatory care with breast cancer stage at diagnosis among medicare beneficiaries. *Journal of General Internal Medicine*.

[17] Gornick ME, Eggers PW, Riley GF (2004). Associations of race, education, and patterns of preventive service use with stage of cancer at time of diagnosis. *Health Services Research*.

[18] Fleming ST, Pursley HG, Newman B, Pavlov D, Chen K (2005). Comorbidity as a predictor of stage of illness for patients with breast cancer. *Medical Care*.

[19] Clegg LX, Reichman ME, Miller BA (2009). Impact of socioeconomic status on cancer incidence and stage at diagnosis: selected findings from the surveillance, epidemiology, and end results: National Longitudinal Mortality Study. *Cancer Causes and Control*.

[20] Warren JL, Klabunde CN, Schrag D, Bach PB, Riley GF (2002). Overview of the SEER-Medicare data: content, research applications, and generalizability to the United States elderly population. *Medical Care*.

[21] National Cancer Institute Overview of the SEER Program. http://seer.cancer.gov/about/overview.html.

[22] National Cancer Institute Overview of the SEER Program. http://healthservices.cancer.gov/seermedicare/overview/linked.html.

[23] The American Cancer Society Cancer Action Network Cancer and Medicare: a chartbook. http://action.acscan.org/site/DocServer/medicare-chartbook.pdf?docID=12061.

[24] Hebert PL, Geiss LS, Tierney EF, Engelgau MM, Yawn BP, McBean AM (1999). Identifying persons with diabetes using medicare claims data. *The American Journal of Medical Quality*.

[25] Fritz A, Ries L *SEER Program Code Manual*.

[26] Klabunde CN, Potosky AL, Legler JM, Warren JL (2000). Development of a comorbidity index using physician claims data. *Journal of Clinical Epidemiology*.

[27] Klabunde CN, Legler JM, Warren JL, Baldwin L-M, Schrag D (2007). A refined comorbidity measurement algorithm for claims-based studies of breast, prostate, colorectal, and lung cancer patients. *Annals of Epidemiology*.

[28] National Cancer Institute Overview of the SEER Program. http://healthservices.cancer.gov/seermedicare/program/remove.ruleout.dxcodes.macro.txt.

[29] National Cancer Institute of the SEER Program. http://healthservices.cancer.gov/seermedicare/program/charlson.comorbidity.macro.txt.

[30] Charlson ME, Pompei P, Ales KA, MacKenzie CR (1987). A new method of classifying prognostic comorbidity in longitudinal studies: development and validation. *Journal of Chronic Diseases*.

[31] Deyo RA, Cherkin DC, Ciol MA (1992). Adapting a clinical comorbidity index for use with ICD-9-CM administrative databases. *Journal of Clinical Epidemiology*.

[32] Romano PS, Roos LL, Luft HS, Jollis JG, Doliszny K (1994). A comparison of administrative versus clinical data: coronary artery bypass surgery as an example. *Journal of Clinical Epidemiology*.

[33] Ashkenazy R, Abrahamson MJ (2006). Medicare coverage for patients with diabetes: a national plan with individual consequences. *Journal of General Internal Medicine*.

